# Mutant IDH1 gliomas downregulate phosphocholine and phosphoethanolamine synthesis in a 2-hydroxyglutarate-dependent manner

**DOI:** 10.1186/s40170-018-0178-3

**Published:** 2018-04-03

**Authors:** Pavithra Viswanath, Marina Radoul, Jose Luis Izquierdo-Garcia, Hema Artee Luchman, J. Gregory Cairncross, Russell O. Pieper, Joanna J. Phillips, Sabrina M. Ronen

**Affiliations:** 1 Department of Radiology and Biomedical Imaging, University of California San Francisco, 1700 4th Street, Box 2532. Byers Hall 3rd Floor, Suite, San Francisco, CA 94143 USA; 2 Centro Nacional de Investigaciones Cardiovasculares (CNIC), Madrid, Spain; 3 CIBER de Enfermedades Respiratorias (CIBERES), Madrid, Spain; 4 Department of Cell Biology and Anatomy and Hotchkiss Brain Institute, University of Calgary, Calgary, Alberta Canada; 5 Department of Clinical Neurosciences and Southern Alberta Cancer Research Institute, University of Calgary, Calgary, Alberta Canada; 6 Department of Neurological Surgery, Helen Diller Research Center, University of California San Francisco, San Francisco, CA USA

**Keywords:** IDH1 mutation, 2-Hydroxyglutarate, Metabolic reprogramming, Magnetic resonance spectroscopy, Phosphocholine, Phosphoethanolamine, Choline kinase, Ethanolamine kinase, HIF-1α, Brain tumors

## Abstract

**Background:**

Magnetic resonance spectroscopy (MRS) studies have identified elevated levels of the phospholipid precursor phosphocholine (PC) and phosphoethanolamine (PE) as metabolic hallmarks of cancer. Unusually, however, PC and PE levels are reduced in mutant isocitrate dehydrogenase 1 (IDHmut) gliomas that produce the oncometabolite 2-hydroxyglutarate (2-HG) relative to wild-type IDH1 (IDHwt) gliomas. The goal of this study was to determine the molecular mechanism underlying this unusual metabolic reprogramming in IDHmut gliomas.

**Methods:**

Steady-state PC and PE were quantified using ^31^P-MRS. To quantify de novo PC and PE synthesis, we used ^13^C-MRS and measured flux to ^13^C-PC and ^13^C-PE in cells incubated with [1,2-^13^C]-choline and [1,2-^13^C]-ethanolamine. The activities of choline kinase (CK) and ethanolamine kinase (EK), the enzymes responsible for PC and PE synthesis, were quantified using ^31^P-MR-based assays. To interrogate the role of 2-HG, we examined IDHwt cells incubated with 2-HG and, conversely, IDHmut cells treated with the IDHmut inhibitor AGI-5198. To examine the role of hypoxia-inducible factor 1-α (HIF-1α), we silenced HIF-1α using RNA interference. To confirm our findings in vivo and in the clinic, we studied IDHwt and IDHmut orthotopic tumor xenografts and glioma patient biopsies.

**Results:**

De novo synthesis of PC and PE was reduced in IDHmut cells relative to IDHwt. Concomitantly, CK activity and EK activity were reduced in IDHmut cells. Pharmacological manipulation of 2-HG levels established that 2-HG was responsible for reduced CK activity, EK activity, PC and PE. 2-HG has previously been reported to stabilize levels of HIF-1α, a known regulator of CK activity. Silencing HIF-1α in IDHmut cells restored CK activity, EK activity, PC and PE to IDHwt levels. Our findings were recapitulated in IDHmut orthotopic tumor xenografts and, most importantly, in IDHmut patient biopsies, validating our findings in vivo and in the clinic.

**Conclusions:**

This study identifies, to our knowledge for the first time, a direct role for 2-HG in the downregulation of CK and EK activity, and thereby, PC and PE synthesis in IDHmut gliomas. These results highlight the unusual reprogramming of phospholipid metabolism in IDHmut gliomas and have implications for the identification of MRS-detectable metabolic biomarkers associated with 2-HG status.

**Electronic supplementary material:**

The online version of this article (10.1186/s40170-018-0178-3) contains supplementary material, which is available to authorized users.

## Background

Mutations in isocitrate dehydrogenase 1 (*IDH1*) are characteristic of low-grade gliomas and secondary upgraded glioblastomas [1]. The wild-type IDH1 (IDHwt) enzyme converts isocitrate to α-ketoglutarate (α-KG), while the mutant IDH1 (IDHmut) enzyme converts α-KG to the oncometabolite 2-hydroxyglutarate (2-HG). By competitively inhibiting the activity of α-KG-dependent dioxygenases such as prolyl hydroxylases, JmJc-domain histone demethylases, and the TET family of 5-methylcytosine hydroxylases, 2-HG induces alterations in epigenetics and cell signaling that ultimately drive tumorigenesis [1, 2].

**Fig. 1:**
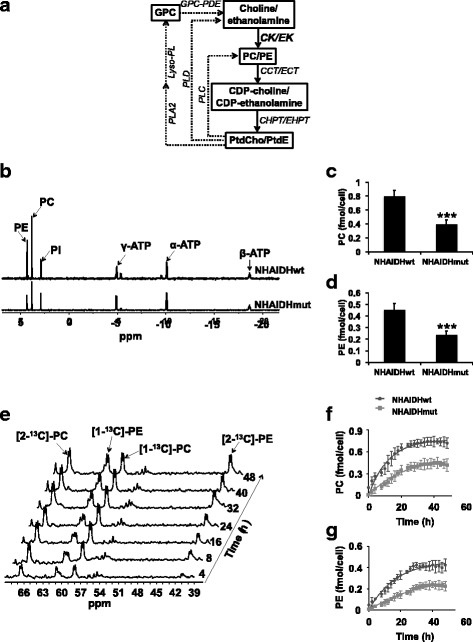
PC and PE synthesis is downregulated in IDHmut glioma cells. **a** Metabolic pathways of choline and ethanolamine phospholipid metabolism in mammalian cells. CK, choline kinase; EK, ethanolamine kinase; CCT, CTP:PC cytidylyltransferase; ECT, CTP:PE cytidylyltransferase; CHPT, choline phosphotransferase; EHPT, ethanolamine phosphotransferase; PLA2, phospholipase A2; Lyso-PL, lyso-phospholipase. Straight lines represent the phospholipid synthetic pathways while dotted lines represent the degradation pathways. **b** Representative ^31^P-MR spectra of the aqueous fraction of cell extracts in the NHA model. Pi, inorganic phosphate; ATP, adenosine triphosphate. PC (**c**) and PE (**d**) levels in the NHA model. **e** Representative ^13^C-MR spectra showing the build-up of [1-^13^C]-PC, [2-^13^C]-PC, [1-^13^C]-PE, and [2-^13^C]-PE over 48 h in live NHAIDHmut cells in an MR-compatible bioreactor. Representative non-linear kinetic fits of de novo synthesis of [1-^13^C]-PC (**f**) and [2-^13^C]-PE (**g**) in the NHA model

The IDH1 mutation also induces metabolic reprogramming that often differs from that observed in IDHwt gliomas [3]. For instance, the Warburg effect, characterized by increased glucose uptake and lactate production, is a metabolic feature of cancer cells, including IDHwt gliomas [4]. However, IDHmut gliomas downregulate lactate production compared to IDHwt [5, 6]. Likewise, elevated phospholipid metabolism is a hallmark of cancer. Phosphocholine (PC) and phosphoethanolamine (PE) are intermediates in the synthesis of phosphatidylcholine (PtdCho) and phosphatidylethanolamine (PtdE), which are quantitatively the most important phospholipids in the cell [7]. Levels of PC, and oftentimes PE, are elevated in every cancer studied to date, including IDHwt gliomas [8, 9]. This effect is typically mediated by overexpression and increased activity of choline kinase (CK) and, less frequently, ethanolamine kinase (EK), the enzymes that phosphorylate choline and ethanolamine to PC and PE, respectively (Fig. 1a) [8–10]. Unusually, however, we and others have previously found that steady-state PC levels are reduced in IDHmut cells relative to IDHwt [11, 12], while Esmaeili et al. showed reduced levels of PE in IDHmut gliomas relative to IDHwt [13].

Magnetic resonance spectroscopy (MRS) is a non-invasive metabolic imaging modality that can be used to quantify metabolism in cells, animals, and patients [14]. ^1^H- and ^31^P-MRS quantify steady-state metabolite levels, whereas ^13^C-MRS monitors metabolic fluxes [15]. ^31^P-MRS can detect PC and PE as well as the phospholipid breakdown products glycerophosphocholine (GPC) and glycerophosphoethanolamine (GPE). ^1^H-MRS detects total choline (tCho)-containing metabolites comprised of choline, PC, and GPC. Elevated levels of PC and tCho can be detected using MRS in most tumors compared to normal tissue, including high-grade IDHwt glioblastomas, and have emerged as non-invasive imaging biomarkers of cancer [16–18]. The reduction in PC and PE observed in IDHmut gliomas was therefore considered as unusual and with potential for serving as an indicator of IDH status [3, 11–13].

The goal of this study was to investigate the molecular mechanism driving the modulation of PC and PE levels in IDHmut gliomas in order to potentially validate these compounds as metabolic imaging biomarkers of the IDH mutation [11–13]. Using genetically engineered and patient-derived cell models as well as orthotopic tumor xenografts, we show that 2-HG production in IDHmut gliomas leads to reduced CK and EK activity and thereby reduced PC and PE synthesis and steady-state levels. Our data indicates that this effect is mediated by hypoxia inducible factor-1α (HIF-1α). Importantly, we confirmed our findings in glioma patient biopsies, highlighting their clinical relevance. Collectively, our study illuminates the mechanism underlying the unusual downregulation of choline and ethanolamine metabolism in IDHmut gliomas and also points to the potential and challenges associated with PC and PE as biomarkers of these tumors.

## Methods

### Cell culture

The generation and characterization of U87 and NHA cells expressing IDHwt (U87IDHwt and NHAIDHwt) or IDH1 R132H mutant enzyme (U87IDHmut and NHAIDHmut) have been previously described [11]. Briefly, all cell lines were maintained between passages 15 and 30 in Dulbecco’s modified Eagle’s medium (DMEM) supplemented with 10% fetal calf serum, 2 mM glutamine, and 100 U/ml penicillin and streptomycin under normoxic conditions. In addition, the medium was supplemented with 28 μM choline (the normal concentration of choline in DMEM is 28 μM and, therefore, the final concentration of choline was 56 μM) and 56 μM ethanolamine in order to mimic the conditions used in our ^13^C-MRS studies (see below). We established that the addition of choline and ethanolamine as described above did not alter the growth as determined by cell counting or proliferation as measured in doubling time of our cells for at least 15 passages. Nor did the addition of choline and ethanolamine alter total PC and PE levels as determined by examination of ^31^P-MRS data from cell extracts as well as bioreactors. The BT54 patient-derived model was provided by H.A.L and J.G.C and cultured as neurospheres in serum-free medium as previously described [19] and the Neurocult medium supplemented with a concentration of 56 μM choline and 56 μM ethanolamine. All cell lines were routinely tested for mycoplasma contamination and authenticated by short tandem repeat fingerprinting (Cell Line Genetics) and assayed within 6 months of authentication.

### Cell treatments

IDHwt and IDHmut siHIF-1α cells were treated with 1 mM D-2-HG (Sigma-Aldrich) for 24 h. U87IDHwt and U87IDHmut siHIF-1α cells were permeabilized with 0.01% digitonin (Sigma-Aldrich) in phosphate-buffered saline (PBS) for 10 min prior to treatment. IDHmut cells were treated with 10 μM AGI-5198 for 72 h. DMSO was used as vehicle control in all cases.

### RNA interference

SMARTpool siRNA (Dharmacon, GE) against human HIF-1α (M-004018-05) and non-targeting siRNA pool #2 (D-001206-14-05) were transfected according to manufacturer’s instructions. Briefly, 2 × 10^6^ U87 or NHA cells, or 2.5 × 10^6^ BT54 cells as neurospheres, were seeded in a 150-mm dish. Twenty-four hours later, cells were transfected with 25 nM siRNA using DharmaFECT 4 transfection reagent (GE) and were incubated at 37 °C for an additional 72 h prior to harvesting for further studies.

### MRS of cell extracts

Metabolite extraction was performed using the dual-phase methanol-chloroform extraction method [11], and spectra were recorded on a 500-MHz spectrometer (Bruker) equipped with a Triple Resonance CryoProbe. ^1^H-MR spectra (1D water presaturation ZGPR sequence, 90° flip angle, 3 s relaxation delay, 256 acquisitions), proton-decoupled ^13^C-MR spectra (30° flip angle, 3 s relaxation delay, 2048 acquisitions) and proton-decoupled ^31^P-MR spectra (30° flip angle, 2.6 s relaxation delay, 1440 acquisitions) were acquired; peak integrals were quantified using Mnova (Mestrelab), corrected for saturation effects, and normalized to cell number and to an external reference (trimethylsilyl propionate for ^1^H- and ^13^C-MR and methylene diphosphonic acid for ^31^P).

### MRS of live cells

^13^C-MRS studies to quantify flux from [1,2-^13^C]-choline and [1,2-^13^C]-ethanolamine to PC and PE in live cells were performed on a 600-MHz Bruker spectrometer using a 10-mm broadband probe as previously described [20]. Cells were grown on fibronectin-coated microcarrier beads (Pronectin-F, Pall Corporation) and assembled into an MR-compatible bioreactor system [20, 21]. Briefly, a perfusion system consisting of one inflow and two outflow lines was combined with a 10-mm NMR tube at the bottom of which the microcarrier beads with cells were placed. This allowed continuous circulation of culture medium (DMEM) in which choline was replaced with 56 μM [1,2-^13^C]-choline (Sigma-Aldrich) to measure PC synthesis, and 56 μM [1,2-^13^C]-ethanolamine (Sigma-Aldrich) was added in order to measure PE synthesis. The incorporation of [1,2-^13^C]-choline and [1,2-^13^C]-ethanolamine into ^13^C-labeled PC and PE was monitored over the course of 48 h. Prior to and at the end of every study, proton-decoupled ^31^P-MR spectra (30° flip angle, 3 s relaxation delay, and 1024 transients) were obtained to confirm cell viability and to verify that steady-state PC and PE levels matched those obtained from cell extracts (within experimental error). Proton-decoupled ^13^C-MR spectra were acquired in 2 h blocks using a 30° flip angle, 6 s relaxation delay, and 2400 transients. Peak integrals were quantified using Mnova (Mestrelab Research), corrected for saturation and NOE, and normalized to cell number and to a metabolite of known concentration (1.59 mM inorganic phosphate for ^31^P-MR spectra and 5 mM [1-^13^C]-glucose added to the medium for this purpose for ^13^C-MR spectra). The build-up of ^13^C-labeled PC and PE over time was analyzed using a non-linear kinetic model (GraphPad Prism) that assumes that PC and PE are primarily generated by choline/ethanolamine phosphorylation [20]. Therefore, the kinetics of PC build-up are expected to follow the equation ^13C^PC(*t*) = *A* (1−*e*^−*kt*^) where ^13C^PC represents ^13^C-labeled PC at time point *t*, *A* represents the asymptotic value of the ^13^C-labeled pool of PC, i.e., the total steady state PC pool, and *k* is the pseudo-first-order rate constant for CK. A similar equation was used to quantify PE build-up and obtain a pseudo-first-order rate constant for EK.

### CK and EK activity

CK activity was determined using ^31^P-MR spectroscopy as described earlier [22]. A similar assay was developed for determining EK activity: cells or tumor tissue were lysed in buffer (10 mM glycylglycine, pH 8, 2 mM dithiothreitol) and lysate added to reaction mix (60 mM glycylglycine, pH 8, 3 mM ethanolamine, 4 mM ATP, and 4 mM MgCl_2_). Proton-decoupled ^31^P-MR spectra (30° flip angle, 2.6 s relaxation delay, 128 transients) were then acquired every 5 min and PE concentration quantified from peak integrals as described above. EK activity was measured by linear regression of the time course of PE production.

### Western blotting

Cells (~ 10^7^) were lysed by sonication in RIPA buffer (25 mM Tris-HCl pH 7.6, 150 mM NaCl, 1% NP-40, 1% sodium deoxycholate, 0.1% SDS) containing 150 nM aprotinin and 1 μM each of leupeptin and E64 protease inhibitor. For HIF-1α analysis, nuclear extracts were prepared using the NE-PER fractionation kit (Thermo-Fisher Scientific) according to manufacturer’s instructions. Lysates were cleared by centrifugation at 14,000 rpm for 15 min at 4 °C and boiled in SDS-PAGE sample buffer (95 °C for 10 min). Total cellular protein (~ 20 μg) was separated on a 10% polyacrylamide gel (Bio-Rad) by sodium dodecyl sulphate polyacrylamide gel electrophoresis, transferred onto Immobilon-FL PVDF membrane (Millipore) and probed for HIF-1α (Cell Signaling, 3716), CKα (Abcam, ab38290), ETNK1 (Thermo-Fisher, PA5-28325), and ETNK2 (Thermo-Fisher, PA5-38807). β-Actin (Cell Signaling, 4970), GAPDH (Cell Signaling, 2118), and β-tubulin (Cell Signaling, 2128) were used as loading control.

### Animal studies

Animal studies were conducted in accordance with the University of California Institutional Animal Care and Use Committee (IACUC) guidelines under protocol number AN101013. U87IDHwt and U87IDHmut cells (3 × 10^5^cells/10 μl) were intracranially injected into athymic nu/nu mice (Simonsen Laboratories) by the free-hand technique. T2-weighted MR imaging used to monitor tumor volume was performed on a 14.1-T vertical MR system (Agilent Technologies) equipped with a single-channel ^1^H coil. Images were acquired using a multislice spin-echo sequence with the following parameters: time-to-echo 20 ms; repetition time 1200 ms; field of view 25 × 25 mm^2^; matrix 512 × 256; slice thickness 1.0 mm; and number of averages 2. Tumor contours in each axial slice were drawn manually, and tumor volume was determined as a sum of the areas multiplied by slice thickness using in-house MR software (SIVIC). When the tumors reached ~ 100 mm^3^, the animals were sacrificed and tumor tissue snap frozen for metabolic and biochemical analysis (*n* = 5 for U87IDHwt, *n* = 7 for U87IDHmut).

### DNA hypermethylation

Analysis of methylation of CHKA, ETNK1, and ETNK2 genes was performed as previously described [6]. Briefly, genomic DNA was isolated; bisulfite was converted using the EZ DNA MethylationKit (Zymo Research) and processed on Infinium Human Methylation 450 bead arrays (Illumina). Probes that were significantly different between IDHwt and IDHmut cells were identified using the Limma (moderated *t* test) approach as described previously [23, 24]. The ratio between methylated probe intensity and total probe intensity, which can be interpreted as the percentage of methylation, was designated as the beta (*β*) value and used to calculate the Δ*β* values (the difference between IDHmut and IDHwt cells) for each gene.

### Patient samples

Flash-frozen human tumor tissue (*n* = 4 IDHwt, *n* = 3 IDHmut), with no patient-identifying information, was obtained in compliance with informed consent policy from the UCSF Brain Tumor Center Biorepository and Pathology Core. The Committee on Human Research at UCSF approved sample use, and the Institutional Review Board at UCSF approved research. Tumors were considered IDHmut if they were immunopositive with the anti-IDH1 R132H antibody (DIA-H09, Dianova) and IDHwt if negative for IDH1 mutation, EGFR amplification, and/or PTEN loss as determined clinically by FISH.

### TCGA data analysis

Mean normalized *z*-scores for *CHKA*, *ETNK1*, and *ETNK2* mRNA expression (RNA Seq V2 RSEM dataset) and DNA methylation (HM450 dataset) were calculated from data for low-grade glioma (grades II and III) patient biopsies deposited in the larger TCGA database (http://cancergenome.nih.gov) and downloaded via the CBio Portal (http://www.cbioportal.org) [25, 26].

### Statistical analysis

All experiments were performed on a minimum of five samples and results presented as mean ± standard deviation. Statistical significance was assessed using an unpaired two-tailed Student’s *t* test assuming unequal variance with *p* < 0.05 considered significant. “*” represents *p* < 0.05, “**” represents *p* < 0.01, and “***” represents *p* < 0.005. NS indicates that there is no statistically significant difference.

## Results

### PC and PE synthesis is downregulated in IDHmut glioma cells

We examined PC and PE synthesis in two cell models, a U87 glioblastoma-based model and an immortalized normal human astrocyte (NHA) model. In each case, the cells were genetically engineered to express either IDHwt or IDH1 R132H mutant enzyme [11]. Using ^1^H-MRS, we previously reported that steady-state PC levels were reduced in IDHmut cells relative to IDHwt [11]. Separately, Esmaeili et al. used ^31^P-MRS to show that steady-state PE was reduced in IDHmut gliomas relative to IDHwt [13]. Here, we used ^31^P-MR analysis of cell extracts (Fig. 1b) to confirm both of these findings in our models. Steady-state PC as well as PE were significantly reduced in IDHmut cells relative to IDHwt in both NHA (Fig. 1c, d) and U87 (Additional file 1: Figure S1A-B) models. We also confirmed that steady-state PC pool sizes as determined by ^31^P-MRS matched the values previously determined by ^1^H-MRS in both our NHA and U87 models (Additional file 1: Figure S1C-D).

We then used ^13^C-MRS to monitor PC and PE synthesis in live cells perfused with [1,2-^13^C]-choline and [1,2-^13^C]-ethanolamine over 48 h (Fig. 1e). This time course was chosen because steady-state pools of PC and PE were filled by 48 h (as determined by comparing to our ^31^P-MRS data, Additional file 1: Figure S1E-H) while breakdown products of PtdCho and PtdE such as ^13^C-GPC and ^13^C-GPE were below detection, indicating that ^13^C-labeled PC and PE was generated primarily de novo from extracellular ^13^C-choline and ^13^C-ethanolamine. The kinetic fits for PC and PE build-up are shown in Fig. 1f, g for the NHA model and in Additional file 1: Figure S1I-J for the U87 model. The pseudo-first-order rate constant for CK dropped significantly by 40% from 0.08 ± 0.007 h^−1^ in NHAIDHwt to 0.05 ± 0.004 h^−1^ in NHAIDHmut and by 39% from 0.1 ± 0.008 h^−1^ in U87IDHwt to 0.06 ± 0.007 h^−1^ in U87IDHmut. Similarly, the pseudo-first-order rate constant for EK decreased significantly by 50% from 0.06 ± 0.005 h^−1^ to 0.03 ± 0.004 h^−1^ in the NHA model and by 40% from 0.08 ± 0.005 h^−1^ to 0.05 ± 0.003 h^−1^ in the U87 model.

### CK activity and EK activity are reduced in IDHmut glioma cells

**Fig. 2:**
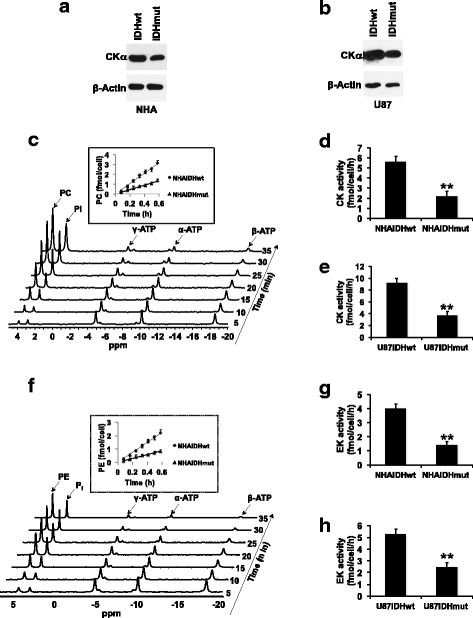
CK activity and EK activity are downregulated in IDHmut glioma cells. CKα expression in the NHA (**a**) and U87 (**b**) models. **c** Representative ^31^P-MR assay for CK activity showing PC build-up following addition of cell lysate to a reaction mix containing choline and ATP. The inset shows the rate of PC production in the NHA model. Quantification of CK activity in the NHA (**d**) and U87 (**e**) models. **f** Representative ^31^P-MR assay for EK activity showing PE build-up following addition of cell lysate to a reaction mix containing ethanolamine and ATP. The inset shows the rate of PE production in the NHA model. Quantification of EK activity in the NHA (**g**) and U87 (**h**) models

Next, we examined the expression and activity of CK and EK, the enzymes responsible for PC and PE synthesis (Fig. 1a) in our models. In mammalian cells, CK exists in three isoforms, CKα1 and CKα2 (encoded by the *CHKA* gene) and CKβ (*CHKB* gene), of which the α but not β isoforms have been associated with malignant transformation [9, 10]. In our IDHmut cells, CKα expression was significantly reduced relative to IDHwt (Fig. 2a, b). Using a ^31^P-MR-based assay (Fig. 2c), we also measured CK activity in cell lysates and found a significant reduction in IDHmut cells relative to IDHwt in both NHA (61%, Fig. 2d) and U87 (60%, Fig. 2e) models.

EK is encoded by the *ETNK1* and *ETNK2* genes [27, 28]. We were unable to detect expression of ETNK1 or ETNK2 using commercially available antibodies in our models (data not shown). Nonetheless, measurement of EK, or EK-like activity in cell lysates (Fig. 2f), showed a significant reduction in IDHmut cells relative to IDHwt in both NHA (65%, Fig. 2g) and U87 (54%, Fig. 2h) models.

### 2-HG mediates the reduction in CK activity and EK activity as well as PC and PE levels

**Fig. 3:**
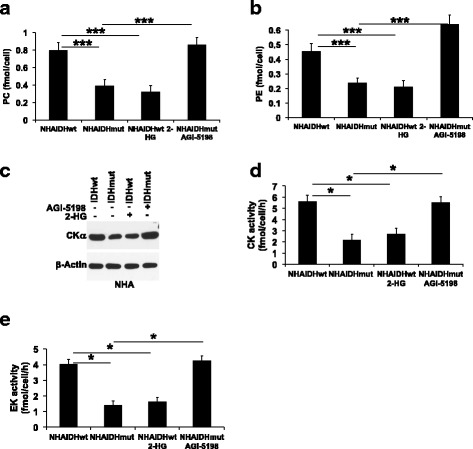
2-HG is responsible for the downregulation of PC and PE synthesis in IDHmut glioma cells. PC (**a**), PE (**b**), CKα expression (**c**), CK activity (**d**), and EK activity (**e**) in NHAIDHwt cells, NHAIDHwt cells incubated with 2-HG, NHAIDHmut cells, and NHAIDHmut cells treated with AGI-5198

In an effort to link our results to the IDH1 mutation, we pharmacologically manipulated the presence of 2-HG and examined choline and ethanolamine metabolism in IDHwt cells incubated with 2-HG or in IDHmut cells treated with the IDHmut enzyme inhibitor AGI-5198 [29, 30]. PC and PE levels were reduced in IDHwt cells treated with 2-HG in both NHA (Fig. 3a, b) and U87 (Additional file 1: Figure S2A-B) models. Conversely, inhibiting 2-HG production by treating IDHmut cells with AGI-5198 restored PC and PE to levels similar to those observed in IDHwt cells. Concomitantly, CKα expression, CK activity, and EK activity were reduced in a manner linked to the presence of 2-HG in both NHA (Fig. 3c–e) and U87 (Additional file 1: Figure S2C-E) models. Collectively, these results mechanistically linked 2-HG to the downregulation of PC and PE synthesis in IDHmut glioma cells.

### HIF-1α downregulates CK and EK activity

**Fig. 4:**
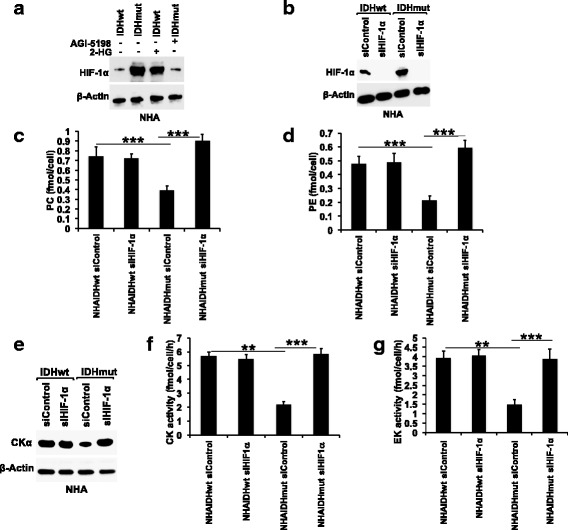
HIF-1α downregulates PC and PE synthesis in IDHmut glioma cells. **a** Representative western blot showing the effect of 2-HG on HIF-1α in the NHA model. **b** Representative western blots showing silencing of HIF-1α in the NHA model. Effect of HIF-1α silencing on PC (**c**), PE (**d**), CKα expression (**e**), CK activity (**f**), and EK activity (**g**) in the NHA model

Next, we looked for the molecular mechanism by which 2-HG downregulates PC and PE synthesis in IDHmut gliomas. HIF-1α has previously been reported to modulate CKα expression and PC levels in cancer [31, 32]. Furthermore, we and others previously demonstrated that HIF-1α levels are post-translationally stabilized in IDHmut gliomas as a result of 2-HG-mediated inhibition of the activity of the prolyl hydroxylases that target HIF-1α for degradation [30, 33–36]. Here, by examining HIF-1α levels in IDHwt cells treated with 2-HG and in IDHmut cells treated with AGI-5198, we confirmed that higher HIF-1α levels are linked to the presence of 2-HG in both NHA (Fig. 4a) and U87 (Additional file 1: Figure S3A) models. We then questioned whether HIF-1α was linked to PC and PE synthesis in our models. To this end, we silenced HIF-1α expression by RNA interference in both IDHwt and IDHmut cells (Fig. 4b and Additional file 1: Figure S3B). Silencing HIF-1α did not alter PC, PE, CKα expression, CK activity, or EK activity in IDHwt cells in both NHA (Fig. 4c–g) and U87 (Additional file 1: Figure S3C-G) models. In contrast, HIF-1α silencing restored PC, PE, CKα expression, CK activity, and EK activity to levels similar to those observed in IDHwt cells in both NHA (Fig. 4c–g) and U87 (Additional file 1: Figure S3C-G) models. We also established that treating IDHmut cells with 2-HG did not rescue the effects of HIF-1α silencing on PC (Additional file 1: Figure S4A-B), PE (Additional file 1: Figure S4C-D), CK activity (Additional file 1: Figure S4E-F), or EK activity (Additional file 1: Figure S4G-H). These results are in line with the observation that 2-HG stabilizes HIF-1α post-translationally [30, 33–36], while siRNA-mediated HIF-1α silencing occurs at the post-transcriptional level [37]. Taken together, these results suggest that 2-HG-mediated stabilization of HIF-1α downregulates CK activity and EK activity and thus leads to reduced PC and PE levels in IDHmut glioma cells.

Finally, 2-HG can alter gene expression in glioma cells via DNA hypermethylation [38, 39]. We, therefore, examined the methylome of IDHwt and IDHmut cells to determine whether the *CHKA*, *ETNK1*, or *ETNK2* genes were hypermethylated in our models. There was no significant difference in the Δ*β* value (difference in % methylation between IDHmut and IDHwt cells) of these genes in our NHA (Δ*β* = 0.02, *p* = 0.4, probe cg27431247 for CHKA, Δ*β* = 0.01, *p* = 0.5, probe cg25881344 for ETNK1, and Δ*β* = 0.03, *p* = 0.2, probe cg08114257 for ETNK2) or U87 (Δ*β* = 0.04, *p* = 0.1, probe cg27431247 for CHKA, Δ*β* = 0.03, *p* = 0.3, probe cg25881344 for ETNK1, and Δ*β* = 0.01, *p* = 0.2, probe cg08114257 for ETNK2) models. These results potentially rule out epigenetic effects of 2-HG on CK and EK expression in our models and suggest that the effect of 2-HG on PC and PE levels is primarily mediated by HIF-1α.

### 2-HG downregulates PC and PE synthesis in the BT54 patient-derived IDHmut glioma model

**Fig. 5:**
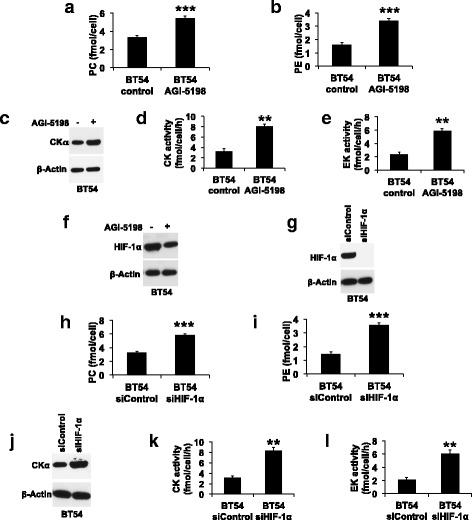
2-HG, acting via HIF-1α, downregulates PC and PE synthesis in the BT54 patient-derived IDHmut glioma model. Effect of AGI-5198 on PC (**a**), PE (**b**), CKα expression (**c**), CK activity (**d**), EK activity (**e**), and HIF-1α (**f**) in the BT54 model. **g** Representative western blots showing silencing of HIF-1α in the BT54 model. Effect of HIF-1α silencing on PC (**h**), PE (**i**), CKα expression (**j**), CK activity (**k**), and EK activity (**l**) in the BT54 model

In order to confirm our findings in a clinically relevant IDHmut glioma model, we examined the BT54 patient-derived neurosphere model [19], which retains both IDHwt and IDHmut alleles and produces 2-HG [5, 19]. Consistent with our results in the genetically engineered models, treating BT54 neurospheres with AGI-5198 increased PC, PE, CKα expression, CK activity, and EK activity (Fig. 5a–e), an effect that was associated with reduced HIF-1α levels (Fig. 5f). Importantly, silencing HIF-1α (Fig. 5g) increased PC, PE, CKα expression, CK activity, and EK activity (Fig. 5h–l), confirming the link between HIF-1α and downregulation of PC and PE synthesis in the clinically relevant BT54 model.

### PC and PE levels are reduced in IDHmut gliomas in orthotopic tumor xenografts

**Fig. 6:**
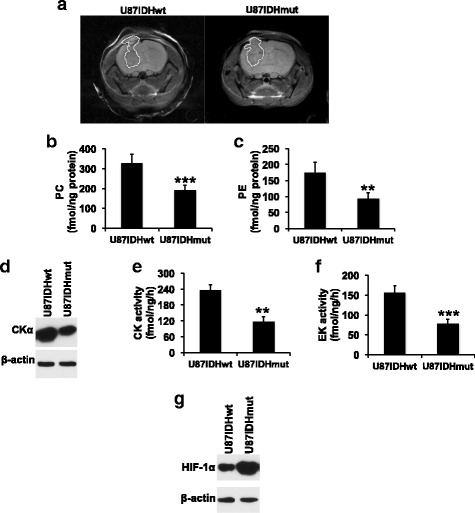
PC and PE synthesis is downregulated in orthotopic IDHmut glioma xenografts. **a** Representative axial T2-weighted MR images of U87IDHwt and U87IDHmut orthotopic tumor-bearing mice. Tumor regions have been manually contoured in white. PC (**b**), PE (**c**), CKα expression (**d**), CK activity (**e**), EK activity (**f**), and HIF-1α (**g**) in U87IDHwt and U87IDHmut tumor xenografts

Next, we examined PC and PE synthesis in U87IDHwt and U87IDHmut orthotopic tumor xenografts. We assessed extracts from tumors that had reached comparable sizes as determined by T2-weighted MR imaging (Fig. 6a). As shown in Fig. 6b, c, PC and PE levels were reduced in U87IDHmut tumors relative to U87IDHwt. Concomitantly, CKα expression (Fig. 6d and Additional file 1: Figure S5A), CK activity (Fig. 6e), and EK activity (Fig. 6f) were reduced in U87IDHmut tumors relative to U87IDHwt. As in the case of our cell models, these metabolic changes were accompanied by higher levels of HIF-1α in U87IDHmut tumors relative to U87IDHwt (Fig. 6g and Additional file 1: Figure S5B).

### PC, PE, CKα expression, CK activity, and EK activity are reduced in IDHmut glioma patient biopsies

**Fig. 7:**
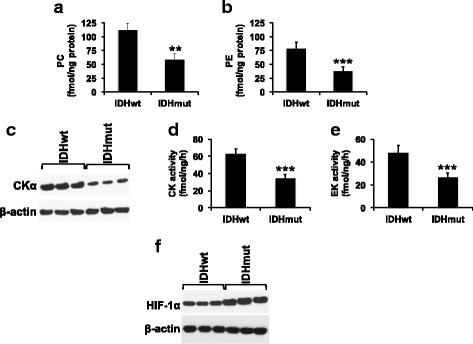
PC and PE synthesis is downregulated in IDHmut glioma patient biopsies. PC (**a**), PE (**b**), CKα expression (**c**), CK activity (**d**), EK activity (**e**), and HIF-1α (**f**) in IDHwt and IDHmut glioma patient biopsies

In order to determine whether our findings were relevant to human patients, we examined IDHwt and IDHmut glioma patient biopsies. Consistent with our results in cell and animal models, levels of PC and PE were reduced in IDHmut glioma patient biopsies relative to IDHwt (Fig. 7a, b). Concomitantly, CKα expression, CK activity, and EK activity were reduced in IDHmut patient biopsies relative to IDHwt (Fig. 7c–e) and this effect was accompanied by an increase in HIF-1α levels in IDHmut biopsies (Fig. 7f).

Finally, we also examined mRNA expression data for the CKα (*CHKA*) and EK genes (*ETNK1* and *ETNK2*) in low-grade (grade II and grade III) patient biopsy samples from The Cancer Genome Atlas (TCGA) database. As shown in Additional file 1: Figure S6A-B, the mean *z*-scores for *CHKA* and *ETNK2* mRNA expression showed a significant reduction in IDHmut glioma samples relative to IDHwt (0.21 in IDHwt to − 0.16 in IDHmut, *p* < 0.0001 for *CHKA* and 0.69 in IDHwt to − 0.12 in IDHmut, *p* < 0.001 for *ETNK2*). There was no significant difference in *ETNK1* mRNA expression (Additional file 1: Figure S6C, 0.11 in IDHwt vs. 0.29 in IDHmut, *p* = 0.2). We also examined DNA methylation for *CHKA, ETNK1*, and *ETNK2* genes within the IDHmut glioma population and, in line with the results in our cell models, found no correlation with mRNA *z*-scores (Pearson correlation coefficients of − 0.2 for *CHKA*, − 0.3 for *ETNK1*, and − 0.3 for *ETNK2*; Additional file 1: Figure S6d-f), suggesting that reduced *CHKA* and *ETNK2* mRNA expression in the IDHmut glioma patient samples was not the result of 2-HG-induced DNA hypermethylation at the promoters of these genes.

## Discussion

In this study, we show that IDHmut gliomas downregulate PC and PE synthesis relative to IDHwt gliomas. This effect is mediated by 2-HG via HIF-1α stabilization and the subsequent downregulation of CK and EK or EK-like activity. Our finding that PC is reduced confirms our previous publication [11], and our results are also in line with the study by Reitman et al. who also showed reduced PC levels in IDHmut glioma cells relative to IDHwt [12]. Esmaeili et al. reported unchanged PC levels in IDHmut gliomas relative to IDHwt [13], but the same study showed a reduction in PE in IDHmut gliomas, consistent with our observations.

PC and PE are precursors in the synthesis of the phospholipids PtdCho and PtdE, which are structural components of cellular membranes [7]. Due to the higher demand for phospholipids associated with cell proliferation, tumor cells often upregulate phospholid biosynthesis [8, 28, 40]. In contrast, we recently demonstrated that 2-HG downregulates PtdCho and PtdE biosynthesis in IDHmut gliomas by inducing autophagic degradation of the endoplasmic reticulum (ER-phagy) [41]. Our observation that PC and PE levels are reduced in IDHmut gliomas is consistent with the reduction in PtdCho and PtdE levels. However, further studies are needed to assess whether the reduction in PC and PE levels induced by HIF-1α is mechanistically linked to the reduction in PtdCho and PtdE levels in IDHmut gliomas and, conversely, whether ER-phagy contributes to the reduction in PC and PE levels in IDHmut gliomas.

We find that reduced CK activity is associated with reduced CKα expression in cell models, tumor xenografts, and patient biopsies. Importantly, CKα (*CHKA*) mRNA expression is reduced in IDHmut patient biopsies relative to IDHwt in the TCGA low-grade glioma dataset. These results are consistent with the central role of CKα in modulating PC levels in cancer [8–10]. CK catalyzes the first committed step in phosphatidylcholine biosynthesis which is the phosphorylation of free choline to PC [10, 42], and studies have shown that this reaction can be rate limiting in cancer cells [42, 43]. With regard to PE, the ethanolamine-specific EK enzymes encoded by the *ETNK1* and *ETNK2* genes mediate PE synthesis in mammalian cells [27, 28]. Although we were unable to detect expression of ETNK1 or ETNK2 in any of our samples using commercially available antibodies, it is important to note that ETNK2 mRNA levels are reduced in IDHmut patient biopsies relative to IDHwt in the TCGA dataset. Further studies are needed to confirm the molecular determinant of PE synthesis in IDHmut gliomas, but it is important to note that CK, which also possesses EK activity, could serve to phosphorylate ethanolamine to PE [9, 10, 44] in IDHmut gliomas.

Our study points to HIF-1α as the primary driver behind reduced CK and EK activity and thus PC and PE levels in IDHmut gliomas. Consistent with our observations, we found no evidence of methylation of the *CHKA*, *ETNK1*, and *ETNK2* genes in our models or in IDHmut glioma patient biopsies in the TCGA dataset, suggesting that 2-HG-induced DNA hypermethylation may not play a major role in downregulating CK and EK activity in IDHmut glioma cells. HIF-1α is a subunit of the HIF transcription factor that regulates expression of several genes involved in tumor proliferation, metabolism, and adaptation to hypoxic stress [45, 46]. In the context of phospholipid metabolism, HIF-1α has been linked to activation as well as repression of CK expression and activity [31, 32]. Furthermore, HIF-1α function can be regulated in a cell type- and context-dependent manner [47]. It is therefore possible that, in IDHmut gliomas, HIF-1α downregulates CK activity. With regard to EK, our study is the first to report downregulation of EK activity by HIF-1α. Further studies are needed to delineate the mechanism by which HIF-1α downregulates CK and EK activity in IDHmut gliomas.

Cellular HIF-1α levels are tightly regulated by post-translational degradation. Under normoxic conditions, HIF-1α is targeted for proteasomal degradation as a result of prolyl hydroxylation that is mediated by α-KG-dependent prolyl hydroxylases [45, 46]. Our finding that 2-HG stabilizes HIF-1α in IDHmut cells, even under normoxic conditions, is consistent with our previous publication [30] as well as previous reports by others indicating that 2-HG competitively inhibits prolyl hydroxylase activity, resulting in HIF-1α stabilization [33–36]. However, other studies have reported unchanged or lower HIF-1α levels in IDHmut gliomas relative to IDHwt [48, 49]. It should be noted that multiple α-KG-dependent prolyl hydroxylases are involved in HIF-1α hydroxylation [50] and the intracellular expression of these enzymes, as well as the efficacy with which 2-HG inhibits these enzymes, together with local levels of hypoxia, may vary, potentially accounting for the discrepancies in HIF-1α expression across different studies.

The complexity of factors affecting HIF-1α levels identifies a potential challenge in using PC and/or PE as imaging biomarkers of IDH status. Another potential confounder that could limit the utility of PC and PE is variability in 2-HG levels. Our results indicate that in IDHmut gliomas PC and PE are directly linked to the ongoing presence of 2-HG. However, a recent study has shown that a small proportion (~ 12%) of IDHmut gliomas spontaneously lose the IDHwt or IDHmut allele, thereby losing the ability to produce 2-HG, an effect that was also associated with progression to a higher grade tumor [51, 52]. Our results suggest that PC and PE synthesis would increase in these tumors. In addition, some ex vivo studies show a correlation between tumor cellularity and PC levels. Accordingly, and consistent with our findings, tissue samples from low-grade (WHO grades II and III) IDHmut gliomas that have undergone malignant progression to a higher cellularity, higher grade (grade IV), glioblastoma show an increase in PC levels and these levels are at least as high as those observed in primary IDHwt glioblastomas [53–55].

In the clinical setting, some in vivo ^1^H-MRS studies suggest that tCho, normalized to creatine or to *N*-acetylaspartate, is higher in higher grade gliomas relative to low-grade [17, 56] and that these ratios could distinguish low-grade IDHmut gliomas from high-grade IDHwt glioblastomas [57, 58]. Most importantly, however, independent of IDH status, when compared to normal brain, normalized tCho is always elevated in the tumor region [59], and thus elevated tCho remains an important and useful indicator of tumor for patients with all brain tumor types [54, 59].

## Conclusions

Our study establishes a role for 2-HG, acting via HIF-1α, in the downregulation of PC and PE synthesis in IDHmut gliomas. These results point to potential challenges in using PC and/or PE as indicators of tumoral IDH status. More importantly, our findings underscore the unusual metabolic reprogramming of IDHmut gliomas [3]. As mentioned, previous studies have identified other metabolic pathways that are reprogrammed differently in IDHmut gliomas compared to IDHwt gliomas [3, 5, 6]. Here, we show, to our knowledge for the first time, oncometabolite-driven downregulation of choline and ethanolamine metabolism in cancer. Our findings thus expand our understanding of the unique metabolic reprogramming associated with the IDH1 mutation.

## Additional file

Additional file 1: **Figures S1-S6.** Provided as a single file containing figures and associated legends. (PDF 895 kb)

## Abbreviations

2-HG: 2-Hydroxyglutarate
CK: Choline kinase
DMEM: Dulbecco’s modified Eagle’s medium
EK: Ethanolamine kinase
GPC: Glycerophosphocholine
GPE: Glycerophosphoethanolamine
HIF-1α: Hypoxia inducible factor-1α
IDH: Isocitrate dehydrogenase
IDHmut: Mutant isocitrate dehydrogenase 1
IDHwt: Wild-type isocitrate dehydrogenase 1
MRS: Magnetic resonance spectroscopy
NHA: Immortalized normal human astrocytes
PC: Phosphocholine
PE: Phosphoethanolamine
tCho: Total choline
WHO: World Health Organization
α-KG: α-Ketoglutarate
