# Supplementary material for: Mutant IDH1 gliomas downregulate phosphocholine and phosphoethanolamine synthesis in a 2-hydroxyglutarate-dependent manner
Source: Cancer Metab. 2018 Apr 3;6:3. doi: 10.1186/s40170-018-0178-3 (PMC5881177; doi:10.1186/s40170-018-0178-3)
Supplement: Supplementary file 1 — Figures S1-S6. Provided as a single file containing figures and associated legends. (PDF 895 kb) [file 40170_2018_178_MOESM1_ESM.pdf]

Supplementary Figure 1

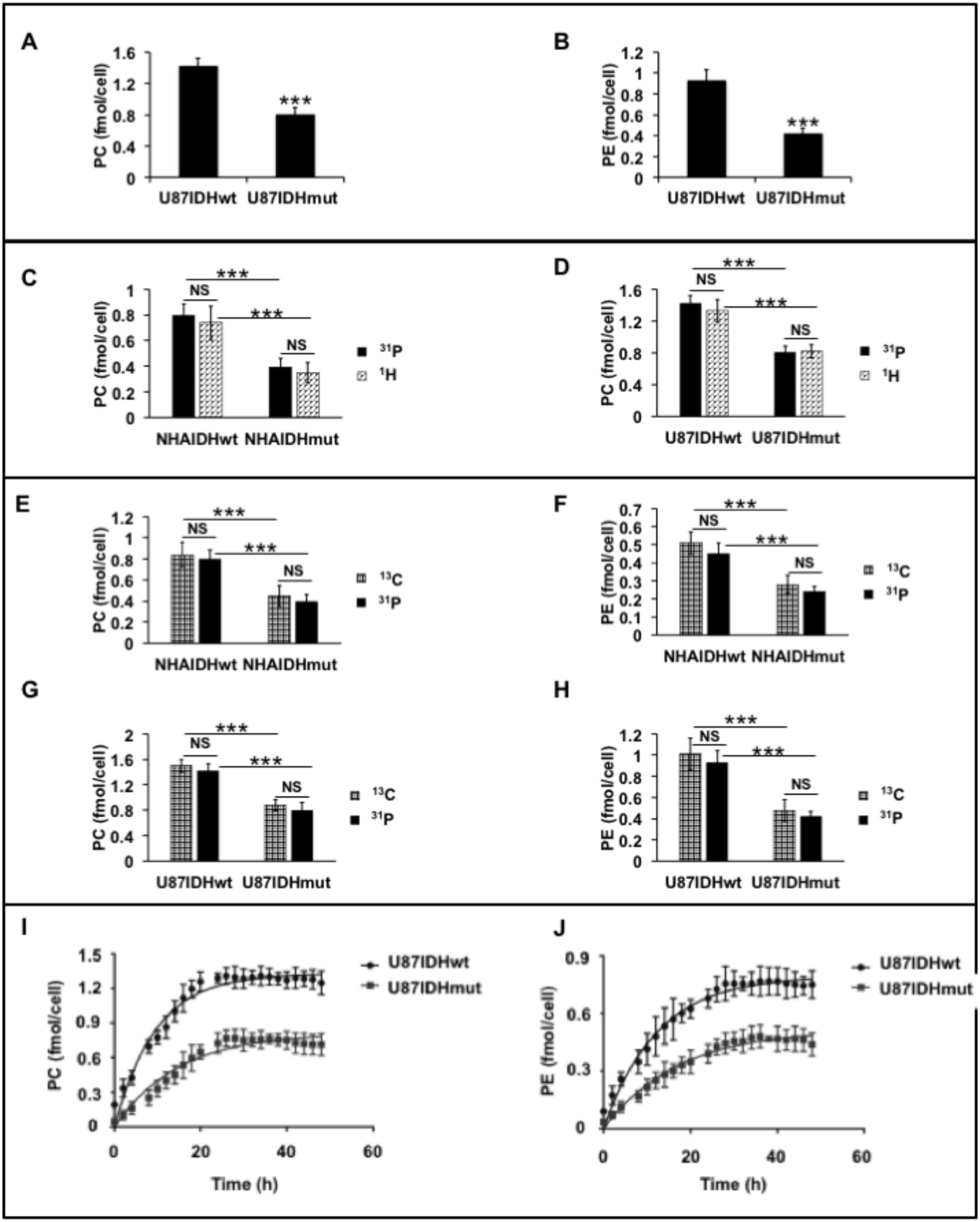

Supplementary Figure 1. PC and PE synthesis is down-regulated in IDHmut glioma cells. PC **(A)** and PE **(B)** levels in the U87 model. Comparison of steady-state PC levels as determined by ^{31}P- and ^{1}H-MRS of cell extracts in the NHA **(C)** and U87 **(D)** models. Comparison of steady-state PC and PE levels as determined by ^{13}C and ^{31}P-MRS of live cells in MR-compatible bioreactors in the NHA **(E,F)** and U87 **(G,H)** models. Representative non-linear kinetic fits of *de novo* synthesis of [1-^{13}C]-PC **(I)** and [2-^{13}C]-PE **(J)** in the U87 model.

Supplementary Figure 2

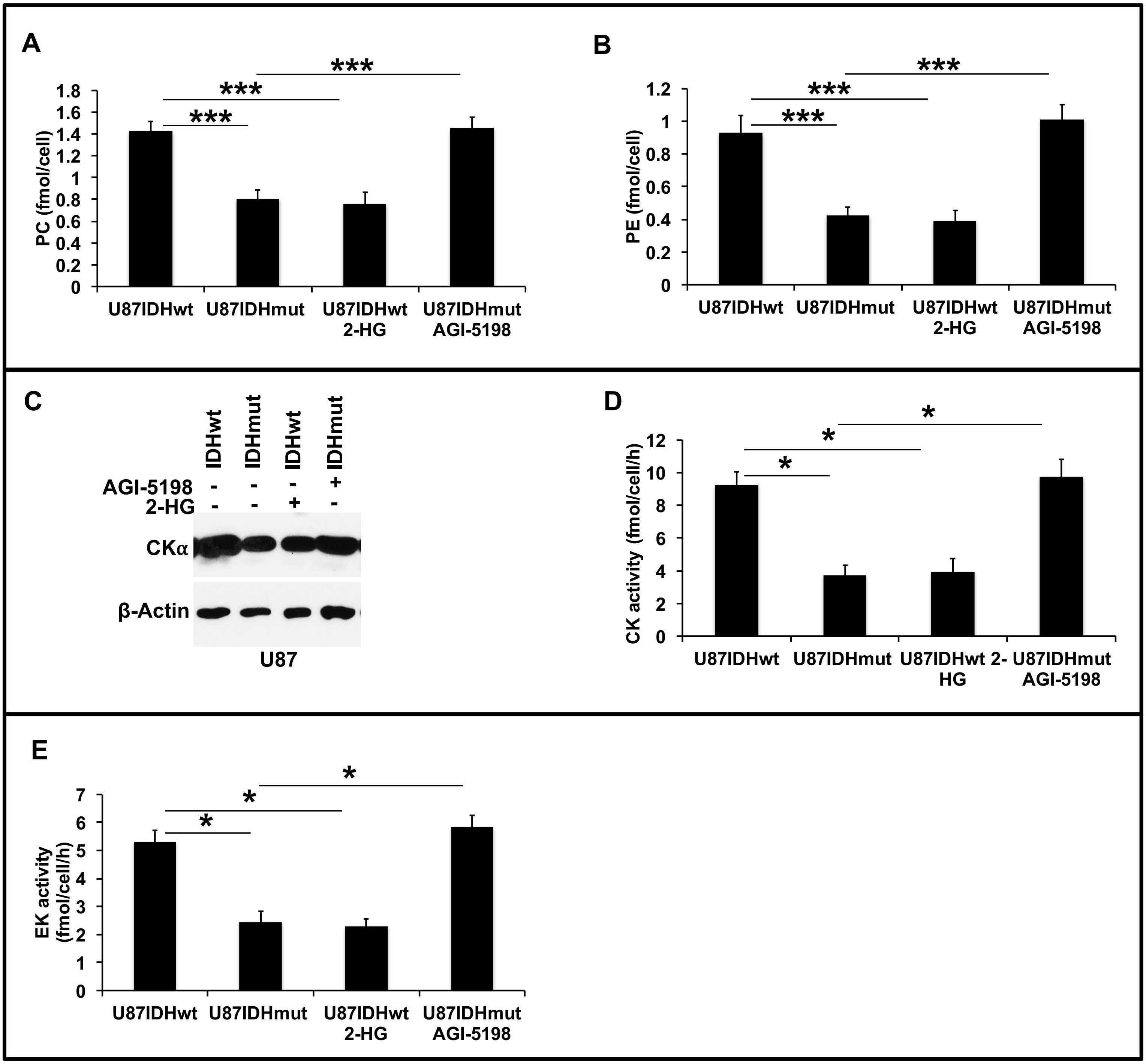

Supplementary Figure 2. 2-HG is responsible for the down-regulation of PC and PE synthesis in IDHmut glioma cells. PC **(A)**, PE **(B)**, CKα expression **(C)**, CK activity **(D)** and EK activity **(E)** in U87IDHwt cells, U87IDHwt cells incubated with 2-HG, U87IDHmut cells and U87IDHmut cells treated with AGI-5198.

Supplementary Figure 3

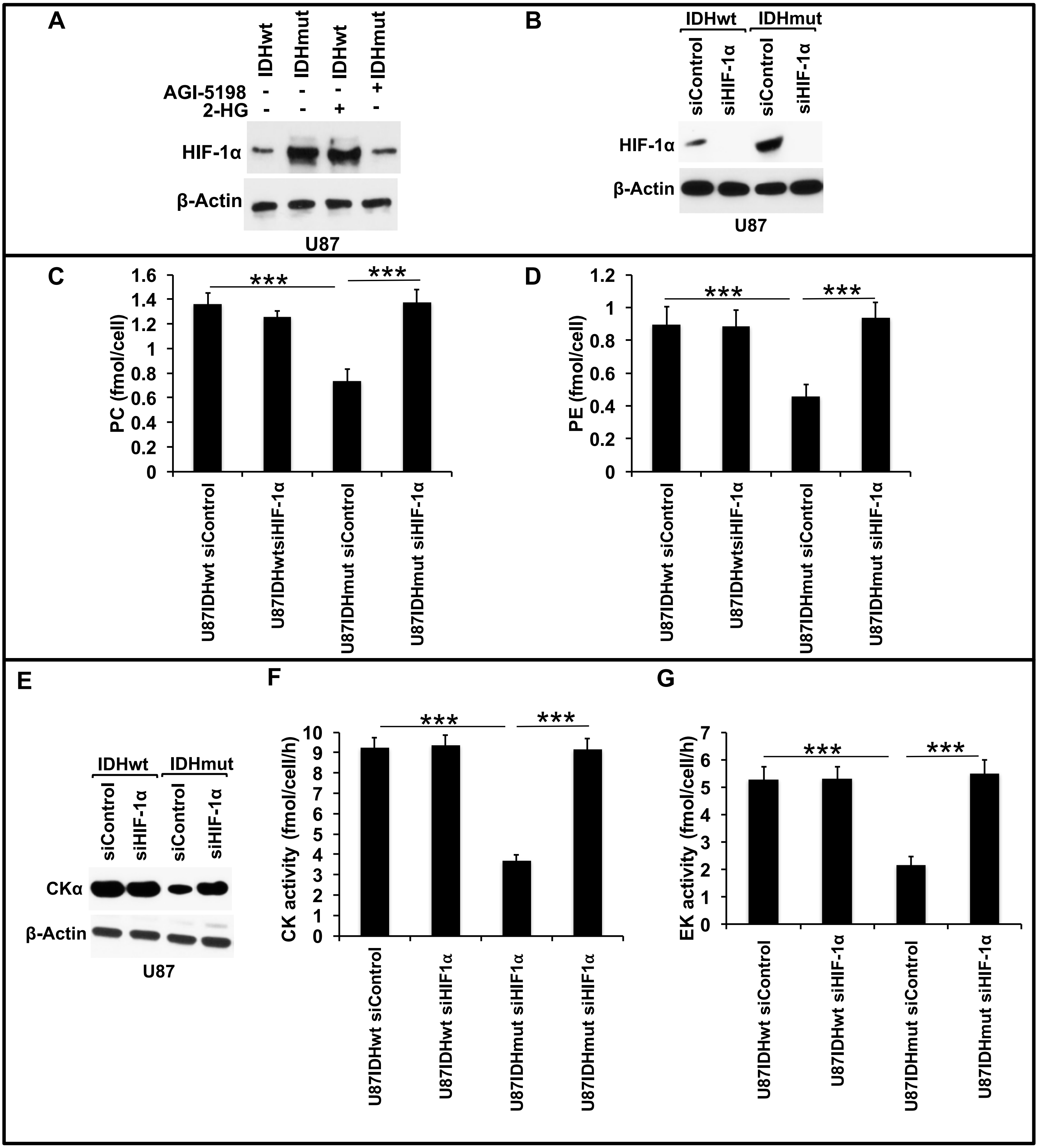

Supplementary Figure 3. HIF-1α down-regulates PC and PE synthesis in IDHmut glioma cells. (A) Representative western blot showing the effect of 2-HG on HIF-1α in the U87 model. **(B)** Representative western blot showing silencing of HIF-1α in the U87 model. Effect of HIF-1α silencing on PC **(C)**, PE **(D)**, CKα expression **(E)**, CK activity **(F)** and EK activity **(G)** in the U87 model.

Supplementary Figure 4

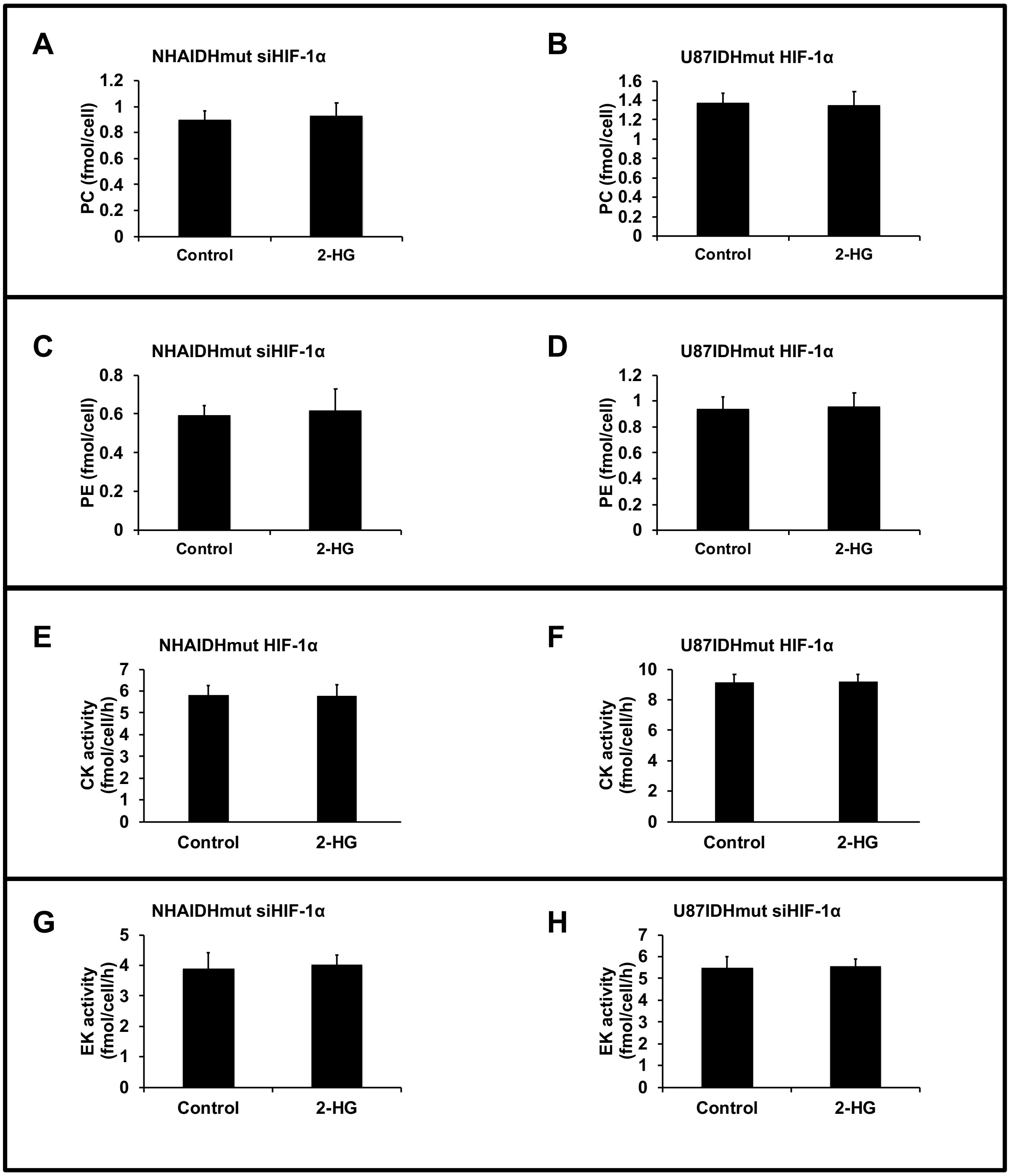

Supplementary Figure 4. 2-HG does not rescue the effects of HIF-1α silencing. Effect of treatment with 2-HG on PC levels in NHAIDHmut siHIF-1α **(A)** and U87IDHmut siHIF-1α **(B)** cells. Effect of treatment with 2-HG on PE levels in NHAIDHmut siHIF-1α **(C)** and U87IDHmut siHIF-1α **(D)** cells. Effect of treatment with 2-HG on CK activity in NHAIDHmut siHIF-1α **(E)** and U87IDHmut siHIF-1α **(F)** cells. Effect of treatment with 2-HG on EK activity in NHAIDHmut siHIF-1α **(G)** and U87IDHmut siHIF-1α **(H)** cells.

Supplementary Figure 5

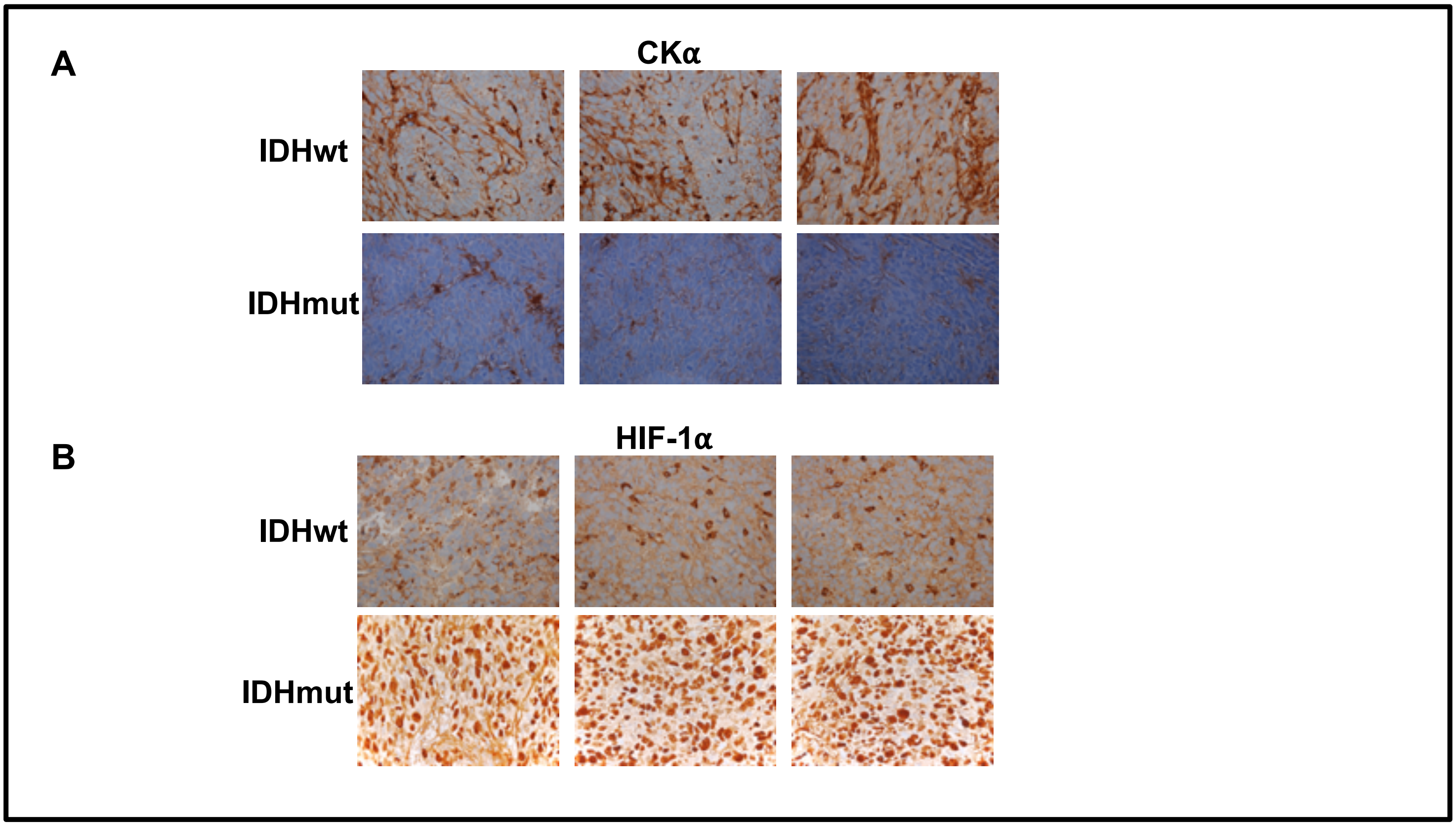

Supplementary Figure 5. U87IDHmut tumor xenografts show higher levels of HIF-1α and lower levels of CKα relative to U87IDHwt tumors. Immunohistochemistry for CKα **(A)** and HIF-1α **(B)** in tissue sections from U87IDHwt and U87IDHmut tumor xenografts.

Supplementary Figure 6

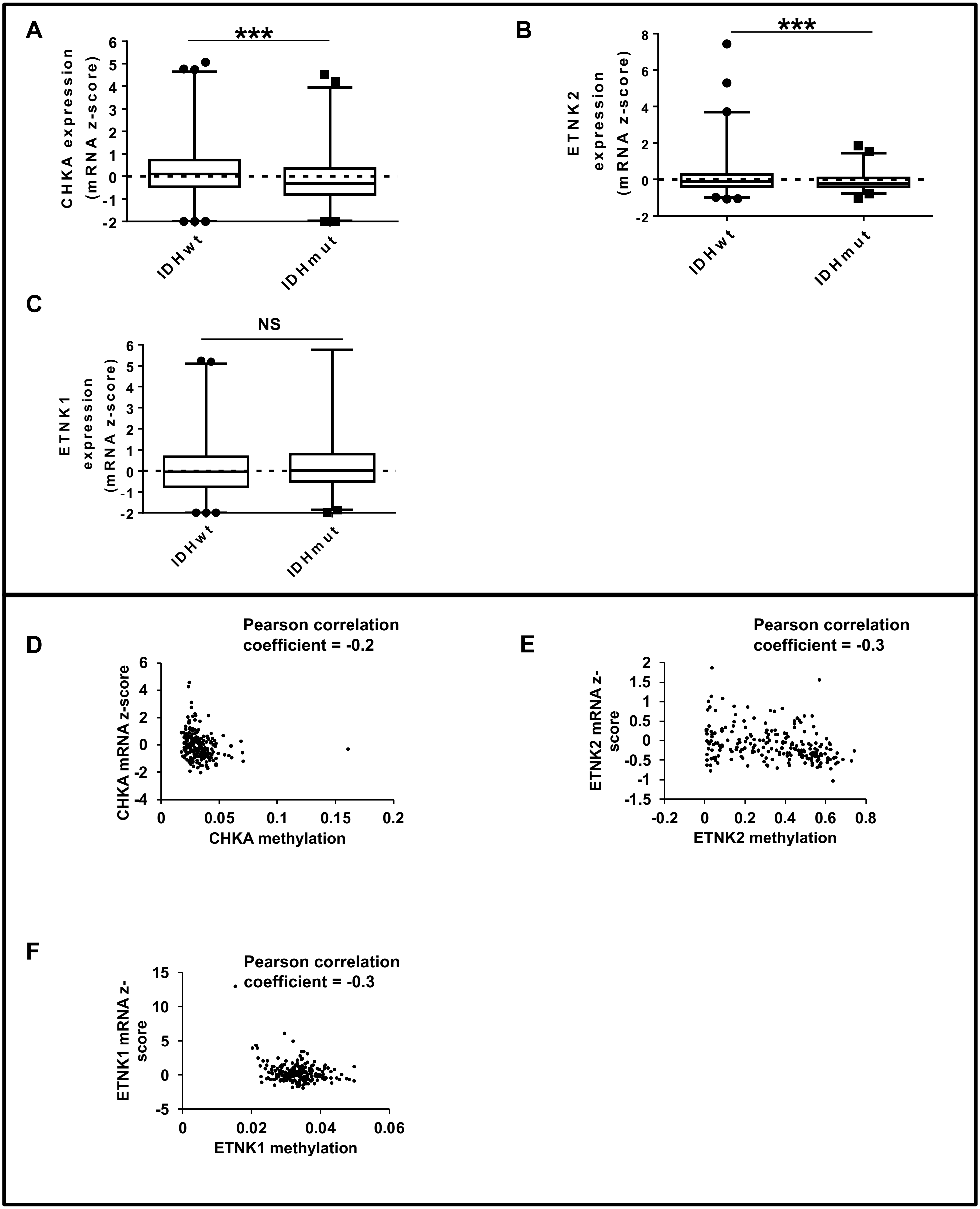

Supplementary Figure 6. *CHKA*, *ETNK1* and *ETNK2* expression are reduced in IDHmut glioma patient biopsies relative to IDHwt. Normalized z-scores for *CHKA* **(A)**, *ETNK1* **(B)** and *ETNK2* **(C)** mRNA expression in IDHmut and IDHwt low-grade glioma patient biopsies as determined by analysis of data deposited in the TCGA database. Boxes denote the mean z-score and whiskers denote the 1-99 percentile for tumors within the group. Correlation between *CHKA* **(D)**, *ETNK1* **(E)** and *ETNK2* **(F)** mRNA z-scores and gene methylation as determined by analysis of data deposited in the TCGA database.
